# Effect of Iron-Oxide Nanoparticles Impregnated Bacterial Cellulose on Overall Properties of Alginate/Casein Hydrogels: Potential Injectable Biomaterial for Wound Healing Applications

**DOI:** 10.3390/polym12112690

**Published:** 2020-11-14

**Authors:** Rahul Patwa, Oyunchimeg Zandraa, Zdenka Capáková, Nabanita Saha, Petr Sáha

**Affiliations:** 1 Centre of Polymer Systems, University Institute, Tomas Bata University in Zlín, Tř. T. Bati 5678, 760 01 Zlín, Czech Republic; zandraa@utb.cz (O.Z.); capakova@utb.cz (Z.C.); saha@utb.cz (P.S.); 2 Faculty of Technology, Tomas Bata University in Zlín, Vavrečkova 275, 760 01 Zlín, Czech Republic

**Keywords:** hydrogel, alginate, bacterial cellulose, drug delivery, magnetic

## Abstract

In this study we report the preparation of novel multicomponent hydrogels as potential biomaterials for injectable hydrogels comprised of alginate, casein and bacterial cellulose impregnated with iron nanoparticles (BCF). These hydrogels demonstrated amide cross-linking of alginate–casein, ionic cross-linking of alginate and supramolecular interaction due to incorporation of BCF. Incorporation of BCF into the hydrogels based on natural biopolymers was done to reinforce the hydrogels and impart magnetic properties critical for targeted drug delivery. This study aimed to improve overall properties of alginate/casein hydrogels by varying the BCF loading. The physico-chemical properties of gels were characterized via FTIR, XRD, DSC, TGA, VSM and mechanical compression. In addition, swelling, drug release, antibacterial activity and cytotoxicity studies were also conducted on these hydrogels. The results indicated that incorporation of BCF in alginate/casein hydrogels led to mechanically stronger gels with magnetic properties, increased porosity and hence increased swelling. A porous structure, which is essential for migration of cells and biomolecule transportation, was confirmed from microscopic analysis. The porous internal structure promoted cell viability, which was confirmed through MTT assay of fibroblasts. Moreover, a hydrogel can be useful for the delivery of essential drugs or biomolecules in a sustained manner for longer durations. These hydrogels are porous, cell viable and possess mechanical properties that match closely to the native tissue. Collectively, these hybrid alginate–casein hydrogels laden with BCF can be fabricated by a facile approach for potential wound healing applications.

## 1. Introduction

During the past few decades, biomaterials have seen a shift from use of artificial materials such as plastics, ceramics and metals to naturally occurring biopolymers such as polysaccharides and proteins [1,2]. Biomaterials at present are used in a variety of forms such as hydrogels, sponges, scaffolds, electrospun mesh and so on [3]. Out of all these, hydrogels have emerged as the most promising candidate for use in biomedical applications such as targeted drug delivery and tissue regeneration due to favorable biocompatibility, biodegradability and low cytotoxicity [4,5]. Hydrogels are essentially porous, with matching physical and mechanical properties to the extracellular matrix (ECM), allowing for the transport of oxygen and moisture, and movement of nutrients, payloads such as drugs, genes and other biomolecules [6]. It is noteworthy to mention that introducing the hydrogel to the target site in the body requires a surgical procedure, which causes pain and discomfort to the patient, is time consuming and is expensive, which ultimately limits the use of hydrogels [7]. These limitations can be overcome with the usage of injectable hydrogels, which bring to the table not only the typical advantages of conventional hydrogels but also target specificity with minimal invasiveness and outstanding shape adaptability [7].

Injectable hydrogels are in-situ forming gels, which upon being injected undergo a sol–gel phase transition by a cross-linking reaction, resulting in a drastic increase in viscosity [8]. Usually, physical cross-linking such as hydrogen bonding, and hydrophobic and electrostatic interactions are preferred routes for gelation. Furthermore, chemical, enzymatic and photochemical routes are also readily used, though there are concerns of toxicity, apoptosis and prolonged irradiation [9,10]. Injectable hydrogels are being used for a plethora of biomedical applications ranging from delivery vehicle for payloads such as genes, nutrients, drugs, etc., for the treatment of a multitude of diseases and cancers to tissue regeneration such as skin, muscles, bones and cartilage [11,12]. They allow for incorporation of fillers ranging from the nano- to micron-size range. Recently, a variety of reinforcements such as zinc-oxide, iron-oxide, graphene-oxide, etc., have been incorporated into hydrogels to induce antibacterial, magnetic and electrical properties, respectively [13,14,15].

Magnetic injectable hydrogels are an important class of hydrogels that provide useful functionalities such as remote actuation by external magnetic field, hyperthermia effect, antimicrobial properties, targeted and controlled delivery, improved thermal properties and tailorable rheological properties, making them suitable candidates for biomedical applications, especially as drug delivery vehicles [16]. Magnetic hydrogels have been reported with different magnetic nanoparticles (MNPs) such as γ-Fe_2_O_3_, Fe_3_O_4_, cobalt ferrite (CoFe_2_O_4_), strontium ferrite (SrFeO19) and so on [14,17,18,19]. Fe_3_O_4_ is a low-cost and abundant MNP with super-paramagnetic and responsive properties, making it a suitable candidate for preparation of magnetic injectable hydrogels. It has been found that the presence of MNPs can play an important factor in cell proliferation [1].

Recently, MNPs have been attached to other filler materials such as silk nanocrystals, cellulose nanocrystals, bacterial cellulose (BC) and so on [1,20,21]. BC has been extensively used as a reinforcing material for biomedical applications over the last few decades, mainly due to high purity, crystallinity, water retention, biocompatibility and mechanical properties [22]. BC is a linear homopolysaccharide where glucan chains (C_6_H_10_O_5_) are linked by hydrogen bonding to form a nano-fibrous network structure. However, under hydrophilic conditions, BC lacks structural integrity, which limits its applications. To overcome this, BC can be modified using its high hydroxyl functionality by carboxylation, esterification, silylation, etc. These chemical modifications require disintegration of BC after fermentation followed by treatment with toxic chemicals, severely affecting biocompatibility and biodegradability. To address this issue, in-situ BC modification has been developed as a single-step approach that involves BC fermentation in the presence of nanoparticles (NPs). Various NPs have been added during BC production, such as carbon nanotubes, graphene oxide, talc, CaCO3, iron-oxide and so on [23,24,25,26]. To the best of our knowledge, studies considering utilization of bacterial cellulose impregnated with iron-oxide nanoparticles for fabrication of magnetic injectable hydrogels have not been investigated until now.

As one of the most used biopolymer in fabricating injectable hydrogel, alginate is a low-cost, linear polysaccharide isolated from brown marine algae. It is water-soluble and consists of β-d-mannuronic and α-l-guluronic acid repeating blocks, which upon interaction with divalent cations such as Ca^2+^, Mg^2+^ and Zn^2+^creates a highly cross-linked gel network. Alginate is known to have excellent properties such as low immunogenicity, gel ability, stability, viscosity, biocompatibility and anti-inflammatory properties, which makes it a favorable material for injectable hydrogels for biomedical applications [27]. It is known that alginate cannot be metabolized by the human body and needs to be cross-linked with other natural biopolymers to promote cell attachment. In addition, alginate standalone hydrogels suffer from inferior mechanical properties such as low tensile modulus and elongation [28,29]. Thus, it is necessary to integrate other biopolymers with alginate to improve its mechanical and cell-adhesion properties. One of these natural biopolymer is casein, which is readily used for the fabrication of hydrogels after combining with other biopolymers such as hyaluronic acid, pectin, etc. [30,31] There have been very few reports on alginate–casein hydrogel systems. Blending casein proteins with alginate has been found to enhance the bioactivity [32,33].

Caseins are protein-based globular biopolymers derived from milk having both hydrophobic and hydrophilic domains. Caseins amount to around 80% of the total proteins available in milk. They have been used in a variety of biomedical applications. They have garnered interest for hydrogel synthesis due to their hydrophilicity, biocompatibility, low toxicity, biodegradability and availability of functional groups for chemical modification [33]. As compared to other available hydrogel proteins, viz. silk fibroin, gelatin, collagen, bovine serum albumin, etc., casein proteins are less expensive and more readily available [30].

In this study, magnetic hybrid hydrogels were prepared that involve chemical interaction between alginate and casein, ionic cross-linking of alginate and interpenetration with bacterial cellulose impregnated with magnetic nanoparticles. The hydrogels were characterized in detail, and it was observed that the interactions led to desirable properties of an injectable hydrogel. The effects of loading of BCF in different proportions on the morphology as well as the swelling, magnetic, compressive and rheological properties of the hydrogels were examined. Minocycline as a model drug was loaded into the hydrogels, and the release profile and antibacterial studies were conducted. Cytocompatibility was assessed using mouse embryonic fibroblast cells to determine the applicability as a suitable transdermal drug delivery system.

## 2. Experimental Methodology

### 2.1. Materials

Sodium alginate, casein (from bovine milk), 1-ethyl-3-(3-dimethylaminopropyl) carbodiimide (EDC), *N*-hydroxy-succinimide (NHS), agar powder, calcium chloride (CaCl_2_), ferrous chloride (FeCl_2_·4H_2_O) (>98%), ferric chloride (FeCl_3_·6H_2_O) (>98%), sodium hydroxide (NaOH) (pellets, >98%) and dimethylformamide (DMF) (>99%) were supplied by Sigma-Aldrich, Praguel, Czech Republic. Ammonium hydroxide (NH_4_OH) (30% solution) was purchased from Fluka, Neu-Ulm, Germany. BC used in this study was produced by strain *Gluconacetobacter xylinus* 3611T procured from Czech Collection of Microorganisms (CCM), Brno, Czech Republic. Minocyclin hydrochloride 50 mg capsules were purchased from Stada Pharma, Prague, Czech Republic. All chemicals were used without any further purification. Double distilled water was used throughout the experimental studies.

### 2.2. Synthesis of Iron-Oxide Nanoparticles (FeNPs)

Magnetite (Fe_3_O_4_) iron-oxide nanoparticles were synthesized by the co-precipitation route with slight modifications to the described methodology [34,35]. Both FeCl_3_·6H_2_O (80 mmol) and FeCl_2_·4H_2_O (40 mmol) were added to a round bottom flask containing 200 mL of anoxic double distilled water. The contents were stirred for 1 h at 200 rpm and maintained at 60 °C under nitrogen flow. Then the stirring speed was increased to 1000 rpm, and chilled NH_4_OH was added dropwise until a constant black precipitate was formed. The contents were stirred for 2 h continuously to allow the reaction to complete. The FeNPs were collected using permanent magnets and given multiple washings with ethanol and distilled water under sonication. Subsequently, they were dried in a lyophilizer and kept in a desiccator until further use.

### 2.3. Synthesis of Bacterial Cellulose Impregnated with Iron-Oxide Nanoparticles (BCF)

**Scheme 1:**
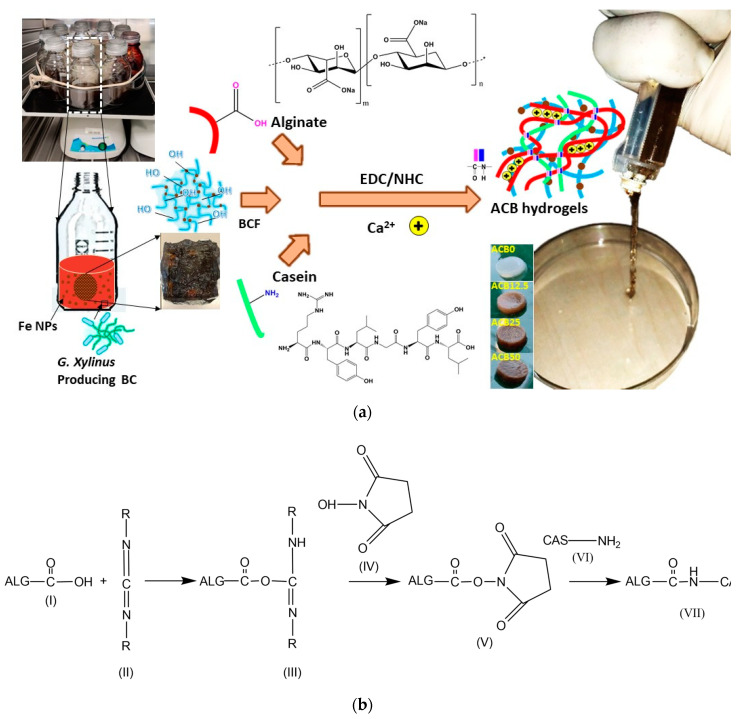
(**a**) Schematic route of preparation of alginate/casein hybrid injectable hydrogels loaded with iron-oxide impregnated bacterial cellulose (BCF); (**b**) reaction pathway for amide bond formation between alginate and casein where (I) alginate, (II) EDC, (III) *O*-acylurea intermediate, (IV) NHS, (V) NHS activated carboxylic group in alginate, (VI) casein and (VII) amide bonds formed.

BCF was synthesized by slight variation of a procedure described elsewhere [24]. First, 250 mL glass bottles containing 100 mL Hestrin–Schramm (HS) media were inoculated with 5 mL of *G. xylinus* 3611 T enriched in HS medium for 2 days at 30 °C. In addition, 3 wt % of Fe NPs were added to each glass bottle, sealed with perforated film for aeration, and incubated at 30 °C for 15 days under nutating motion at ~10 rpm, as shown in Scheme 1a. The iron nanoparticles were taken up by the growing BC fibers resulting in a BCF pellicle. Furthermore, the BCF was suspended in distilled water overnight followed by treatment with 20% NaOH at 80 °C for 1 h. The treated BCF was washed with distilled water until neutral pH and was ground in a Nutribullet high speed mixer (Delimano, Prague, Czech Republic) at 30,000 rpm. The BCF was freeze-dried and kept in a desiccator until further use.

### 2.4. Preparation of Water Soluble Casein.

Water soluble casein was prepared as per the procedure mentioned by Khramtsov et al. [36] by adding casein powder to 1M NaOH to prepare 5% (*w/w*) suspension. The mixture was stirred overnight at ~100 rpm in a beaker to obtain a transparent solution. The solution was dialyzed against 0.15 M NaCl for 6 h followed by distilled water until neutral pH is obtained. The solution was centrifuged at 10,000 rpm for 5 min, and the supernatant was freeze-dried and stored in a desiccator.

### 2.5. Synthesis of BCF Loaded Alginate/Casein Injectable Hydrogels

Firstly, 2 *w/v* % alginate solution was formed by completely dissolving calculated amounts of alginate powder in dH_2_O. Similarly, 2 *w/v* % casein solution was prepared by adding weighted amounts of freeze-dried casein powder in distilled water. Further, both these solutions were mixed, and BCF was then added in different weights to the reaction mixture under gentle stirring ratios, as shown in Table 1. To each batch, EDC and NHS were added in the ratio of 2:2:1 (alginate:EDC:NHS) and allowed to react for 24 h at ambient temperature, as shown in Scheme 1b. Subsequently, the solution was poured into molds made up of 1% agar and 0.05 M CaCl_2_ and kept overnight to complete ionic cross-linking. The formed hydrogels were rinsed with distilled water to remove the chemicals and freeze-dried to form lyophilized gels. Similarly, to form injectable hydrogels, the precursor solution containing all components, viz. alginate, casein, BCF, EDC and NHS, were aspirated inside one cylinder of the double barrel syringe (Sulzer, Risch-Rotkreuz, Switzerland), while 0.05 M CaCl_2_ was filled in another cylinder. Upon injecting, both solutions met at the exit, and the precursor solutions gelled instantly due to ionic cross-linking.

### 2.6. Instrumentation and Characterization Methodology

#### 2.6.1. Fourier Transformed Infrared Spectroscopy

To understand the physico-chemical interaction of individual constituents, ACB hydrogels with or without BCF were examined using a Nicolet–320 Fourier transformed infrared (FTIR) spectrophotometer (ThermoScientific, Atkinson, NH, USA) in attenuated total reflectance (ATR) mode using Ge crystal. Absorption spectra were recorded from 600–4000 cm^−1^ at ambient temperature with a scan rate and resolution of 16 and 4 cm^−1^, respectively, for all materials.

#### 2.6.2. X-ray Diffraction Studies

X-ray diffractograms of individual constituents and hydrogels were recorded using a MiniFlex-600 X-ray diffractometer (XRD) (Rigaku, Tokyo, Japan) with cobalt as an X-ray source. Diffraction patterns were recorded from 2θ, range 5°–75°, with operation voltage, operation current, step time and step size set to 40 kV, 15 mA, 10°/s and 0.02°, respectively. The diffractograms 2θ values were converted to a copper X-ray source using PowerDLL 2.93 software to be able to compare with data in prior studies.

#### 2.6.3. Morphological and Elemental Analysis

The morphology of iron-oxide nanoparticles, synthesized BCF and lyophilized spumous hydrogels were observed by a Nova-450 field emission scanning electron microscope (FE-SEM) (FEI, Hillsboro, OR, USA) using a TLD detector with operation voltage set at 5 kV. The FeNP suspension (0.001%) was drop cast, whereas hydrogel cross-sections were placed on double-sided carbon tape. Conductive gold coating (~120 s) was done prior to examination using a sputtering instrument. The elemental composition of FeNPs and BCF mapping was carried out using Octane plus energy dispersive X-ray (EDX) spectroscopy (EDAX, Ametek Inc., Berwyn, PA, USA).

#### 2.6.4. Mechanical Compression Analysis

Compression tests were performed with swollen hydrogels on a M350-5CT universal tensile testing machine (UTM) (Testometric, Rochdale, England) fitted with a 10 Kgf load cell with a crosshead speed of 1 mm·min^−1^. Cylindrical samples with diameters of 20 mm were used for the analysis, and tests were performed in triplicate. Compression modulus was determined, and mean values with standard deviation were reported.

#### 2.6.5. Swelling Properties

The swelling behavior of magnetic injectable hydrogels was studied using the gravimetric method. The lyophilized hydrogels (25 mm diam) were weighed and immersed in de-ionized water for a total duration of 240 min in a hot-air oven set at 37 °C. After regular intervals, the hydrogel samples were withdrawn, blotted using filter paper, weighed and returned back to the solution. The swell tests were performed in triplicate, and mean with standard deviation were reported as swelling ratio percentage (*SR%*) according to Equation (1).

(1) $$SR\%=\;\left(W_{s}-W_{d}\right)/W_{d}\times 100$$

where, *W*_s_ and *W*_d_ are swollen and dry weights, respectively, of ACB hydrogels.

#### 2.6.6. Thermogravimetric Analysis

Iron loading in BCF and thermal stability analysis of prepared BCF and ACB hydrogels were performed on a Q500 thermogravimetric analyzer (TGA) (TA instruments, New Castle, DE, USA). Each sample (~9 mg) was heated in an alumina pan from 25 to 600 °C at a ramp of 10 °C/min^−1^ under nitrogen gas flow of 100 mL·min^−1^.

#### 2.6.7. Viscoelastic Properties

The viscoelastic measurements of swollen ACB hydrogel samples (20 mm diameter) were conducted using an MCR-502 rotational rheometer (AntonPaar GmbH, Graz, Austria) fitted with a parallel plate of 20 mm diameter. Dynamic frequency sweep tests were performed at 25 °C in oscillation mode in the frequency range of 0.1 to 100 rad·s^−1^ and 1% amplitude strain. The effect of BCF loadings on viscoelastic properties such as loss modulus (G″), storage modulus (G′) and complex viscosity (η^*^) was determined and calculated using Equation (2).

(2) $$\eta^{*}={\left[{\left(\frac{G^{\prime }}{\omega }\right)}^{2}+{\left(\frac{G^{\prime \prime }}{\omega }\right)}^{2}\right]}^{0.5}$$

#### 2.6.8. Differential Scanning Calorimetry

To determine the compatibility amongst different constituents and decomposition temperature of the hydrogels, they were subjected to DSC 1 Star^®^ System differential scanning calorimeter (DSC) (Mettler Toledo, Greifensee, Switzerland). Samples (6.5 ± 1.0 mg) were sealed in an aluminum pan under nitrogen flow of 50 mL·min^−1^ and subjected to heating from 30 to 350 °C at a rate of 10 °C·min^−1^.

#### 2.6.9. Drug Release Studies

The in vitro dug release was performed as per the already reported procedures with slight modification [1]. The hydrogel samples were loaded with the drug minocycline hydrochloride (MH) by soaking in 10 mL drug solution of ~0.5 mg·mL^−1^ in dH_2_O:DMF (93:7) for 48 h at room temperature. The amounts of drugs in the solutions were quantified using an ES-290 UV–vis spectrophotometer (Esse3 SRL, Rettore, Italy) at the wavelength of 345 nm. The lyophilized MH-loaded hydrogels were placed in 30 mL of dH_2_O:DMF (93:7). At regular intervals, 3 mL solution was carefully withdrawn, and the concentration of drug released from hydrogels was measured using UV–vis absorption at 345 nm. The solution was again added back to the bulk solution in order to maintain constant release medium volume. The amounts of MH released was determined from the calibration curve, and the cumulative drug release percentage (CD%) was determined according to Equation (3).

(3) $$CD\%=\;\frac{D_{t}}{D_{0}}\;\times 100$$

where *D_t_* is total amount of MH release at time *t*, and *D_0_* is the initial amount of MH in the hydrogels.

#### 2.6.10. Antibacterial Activity

The antibacterial activity of ACB gels was assessed against *Escherichia coli* (CCM code 4517) and *Staphylococcus aureus* (CCM code 4516) using an agar disc diffusion test. A 100 μL bacterial solution containing an approximate concentration of 12 × 10^8^ cells/mL was uniformly spread on a nutrient agar plate, and drug-loaded hydrogel discs (6 mm) were placed on the top of the agar plates. The plates were then incubated at 37 °C, and incubation zones were recorded after a period of 24 h, 48 h, 1 week and 2 weeks.

#### 2.6.11. In-Vitro Cytocompatibility Studies

To evaluate the cytocompatibility, murine embryonic fibroblasts (ECACC 93061524, England) were used as representative cell lines for initial cytotoxicity screening. Dulbecco’s modified Eagle’s medium (DMEM) (Biosera, Nuaille, France) fortified with 10% fetal bovine serum (FBS) (Biosera, France) and 100 Units per mL penicillin/streptomycin (Biosera, France) was used as culture medium. The prepared hydrogel discs (~15 mm diam) were sterilized by UV–radiation for 30 min and were swollen in culture medium for 2 h. It is noteworthy to mention that sterilization through UV irradiation did not affect the structure of the already cross-linked ACB hydrogels, which was confirmed by visual inspection as well. Subsequently, extracts were prepared according to the EN ISO 10993-12 standard in a concentration of 0.1 mg·mL^−1^ of culture medium and were used within 24 h. Cells were seeded in a concentration of 1 × 10^5^ per mL, and the next day extracts were added (concentration 100%, 75% and 50%) to cells and incubated with cells for 24 h at 37 °C. All assays were performed in quintuplicate, and average values with standard deviation were reported. The cell viability was measured using a tetrazolium-based MTT cell proliferation assay kit (Duchefa Biochemie, Haarlem, Netherlands). The absorbance was measured at 570 nm on an Infinite^®^ 200 PRO plate reader (Tecan, Männedorf, Switzerland). The results were reported as reduction in cell viability in relative terms against cells cultivated in media without extracts, i.e., 1 meaning 100% viability. According to the EN ISO 10993-5 standard, the samples are considered to be non-cytotoxic when they can reach cell viability higher than 80%.

## 3. Results and Discussion

### 3.1. Synthesis of Iron-Oxide Nanoparticles (FeNPs) and Bacterial Cellulose Impregnated with Iron-Oxide Nanoparticles (BCF)

**Figure 1:**
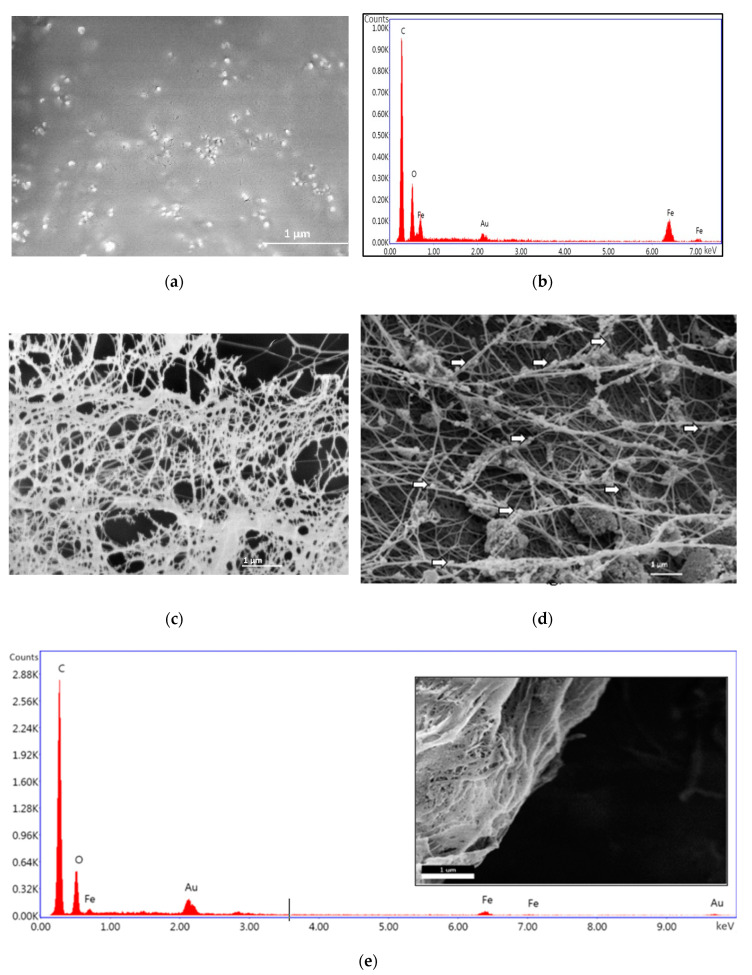

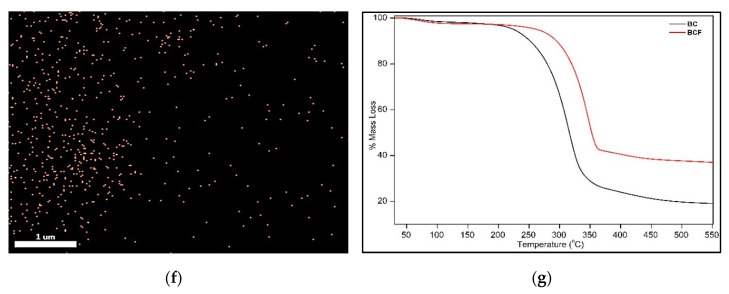
(**a**) FE-SEM micrographs of synthesized FeNPs, (**b**) EDS spectrum of FeNPs displaying elemental information (inset: point selected for EDAX), (**c**) FE-SEM micrograph of BC, (**d**) FE-SEM micrograph of synthesized BCF (FeNPs marked with white arrows), (**e**) EDS spectrum of BCF displaying elemental information, (inset. area selected on BCF specimen for EDS mapping; (**f**) EDS map of FeNPs on BCF surface and (**g**) TGA profiles of BC and BCF.

Figure 1a shows the microscopic image of synthesized iron-oxide nanoparticles (FeNPs) formed by the co-precipitation approach. The nanoparticles had a spherical morphology with average particle size of 47.2 ± 13.7 nm. As can be seen from the micrograph, the nanoparticles tended to form agglomerates consisting of 2–3 particles (shown in black dashed circles), which increased the overall particle size. The point elemental analysis (Figure 1b) showed the presence of iron and oxygen, which revealed that the FeNPs did not contain any other impurities. To confirm the impregnation of FeNPs into the BC, the morphology of both BC and BCF was compared. It was found that BC was composed of a network of nanofibrils (Figure 1c), whereas the BCF morphology (Figure 1d) showed that spherical FeNPs (shown in white arrows) were attached to the BC nanofibrils and homogeneously dispersed throughout the network. Similar findings were reported by Pinto et al. [37,38] The area selected for elemental analysis shown in Figure 1e revealed the presence of iron and oxygen in the BCF (shown in inset). To confirm that the spherical nanoparticles found embedded inside the bacterial cellulose network were iron species, elemental mapping was done (Figure 1f) and it revealed that the iron nanoparticles (Figure 1f, shown in orange) were uniformly dispersed throughout the BCF, which confirmed the synthesis of in situ formation of BCF under dynamic conditions. To estimate the percentage impregnation of iron-oxide into BC, thermogravimetric analysis on BC and BCF was carried out, and it was found that ~20 wt % FeNPs could be loaded onto BC fibrils, as shown in Figure 1g. In addition, BCFs were thermally more stable than BCs.

### 3.2. Morphological Analysis of Alginate–Casein-FeNP-Impregnated Bacterial Cellulose (ACB) Hydrogels

**Figure 2:**
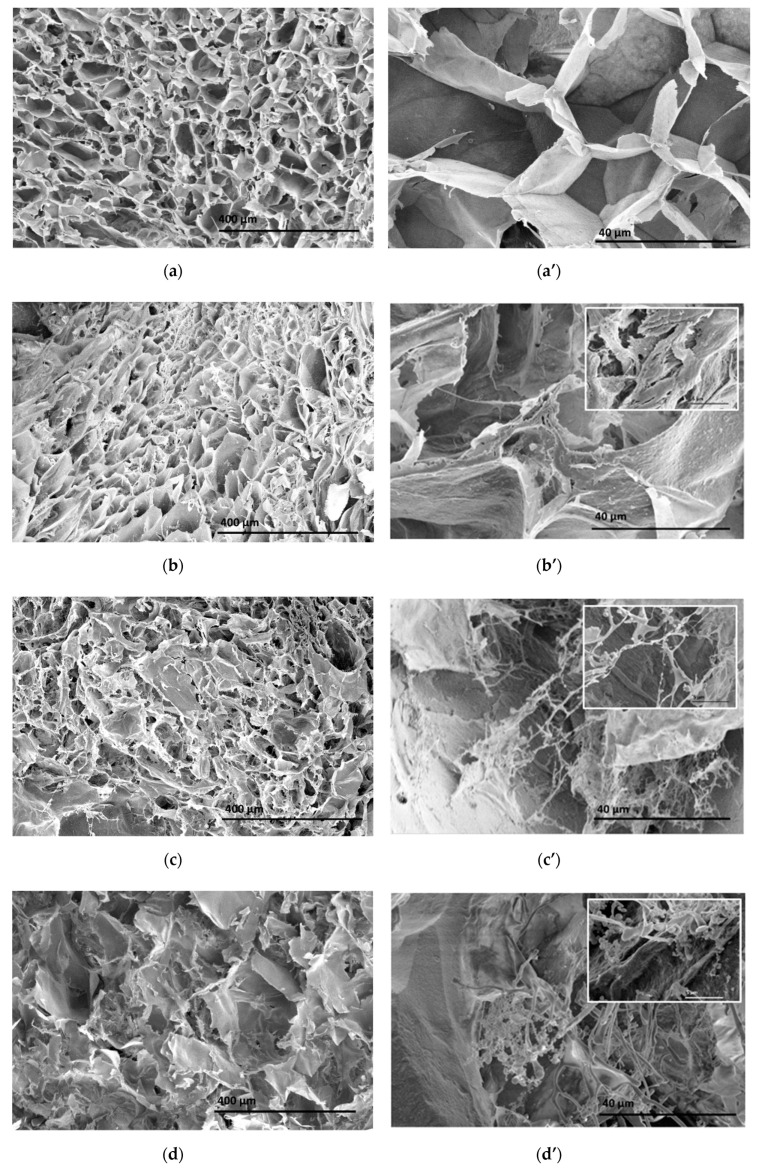
FE-SEM micrographs at 200x, 2000x and 10,000x (shown in inset) for prepared ACB hybrid hydrogels (**a**,**a’**) ACB0, (**b**,**b’**) ACB12.5, (**c**,**c’**) ACB25 and (**d**,**d’**) ACB50 (BCF agglomerate marked with white dashed circle).

The cross-sectional morphologies of the freeze-dried ACB hydrogels were investigated, and SEM micrographs are shown in Figure 2. The micrographs were captured at 200×, 2000× and 10,000× to observe pore structure and distribution of BCF network inside the hydrogels. ACB0 hydrogels (Figure 2a,a’) showed smooth, micron-sized, regular and straight channel arrangements of honeycomb-like pores. Such an open pore structure allows for cells to migrate and differentiate within the hydrogels. However, with the incorporation of BCF in ACB12.5 (Figure 2b,b’) and ACB 25 (Figure 2c,c’), the pore structure became denser and rougher because of the fibrous structure of BCF. The inset images reveal that the alginate–casein biomaterials formed a layer around the BCF network structures, which gave the BCF fibers a smooth appearance. This suggests that the BCF fibrils were uniformly distributed throughout the alginate–casein network. In the case of ACB 50 (Figure 2d,d’), with an even higher loading of BCF, as high as 50 wt %, BCF tended to form agglomerates, which led to highly irregular pores with larger pore sizes due to high loading of BCF. Similar observations on loading of bacterial cellulose of BC and an alginate-based network were previously reported by Taokaew et al. [39].

### 3.3. Swelling Properties of Alginate–Casein-FeNP-Impregnated Bacterial Cellulose (ACB) Hydrogels

**Figure 3:**
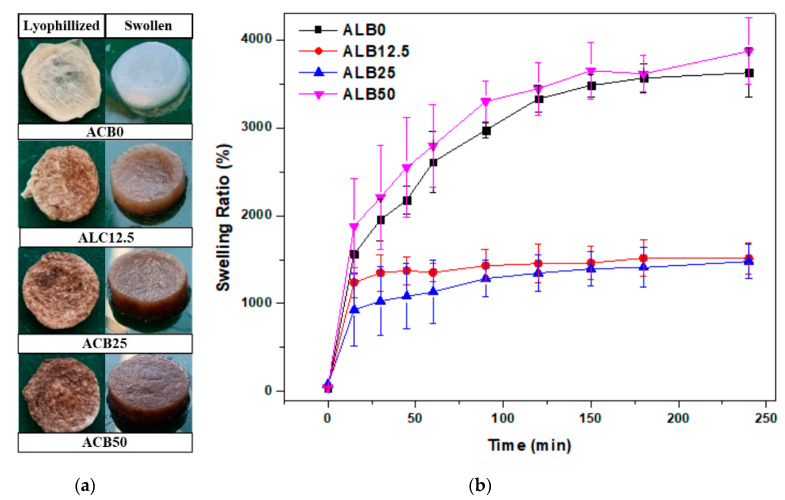
(**a**) Pictorial comparison between ACB hydrogels with different BCF loadings before and after swelling; (**b**) swelling kinetics of the ACB hydrogels as a function of time.

Water absorption is an important parameter for the suitability of a hydrogel for wound dressing. It should be capable of absorbing exudates for a long time whilst still maintaining the necessary structure. Furthermore, it should be able to carry large amounts of payload in the form of nutrients, drugs, cells, genes, etc. Figure 3a, shows images of ACB hydrogels in freeze-dried and swollen states, which indicate that the gels were quite stable even in the swollen state and were able to maintain their structure. In addition, the ACB0 hydrogels were mostly clear and transparent with slight haze, while the hydrogels acquired a blackish/brownish appearance upon incorporation of BCF and were completely opaque. Figure 3b shows the results of lyophilized hydrogels swollen in dH_2_O as a function of time to reach equilibrium water uptake.

For all ACB hydrogels, equilibrium was reached within 180 min of soaking. The ACB0 showed an equilibrium swell percentage of 3567.6% ± 168.2%. However, decreases in ACB12.5 and ACB25 hydrogel water uptake was observed to be 1517.3 ± 203.6% and 1411.4 ± 223.0%, respectively. The plausible reason for this reduction in swelling capacity could be due to hydrogen bonding of BCF with the hydrogel network. These findings were consistent with those of Chunshom et al. and Zhang et al., where increased cross-linking reduced water uptake capacity of alginate-based hydrogels fabricated for facial masks and wound dressing, respectively [40,41]. However, upon further increased loading of BCF to 50 wt %, the swelling percentage drastically improved, as the pores became larger and more irregular (as observed from SEM micrographs shown in Figure 2d), which allowed for more water molecules to penetrate the structure.

### 3.4. Infrared Spectroscopy Studies of Alginate–Casein-FeNP-Impregnated Bacterial Cellulose (ACB) Hydrogels

**Figure 4:**
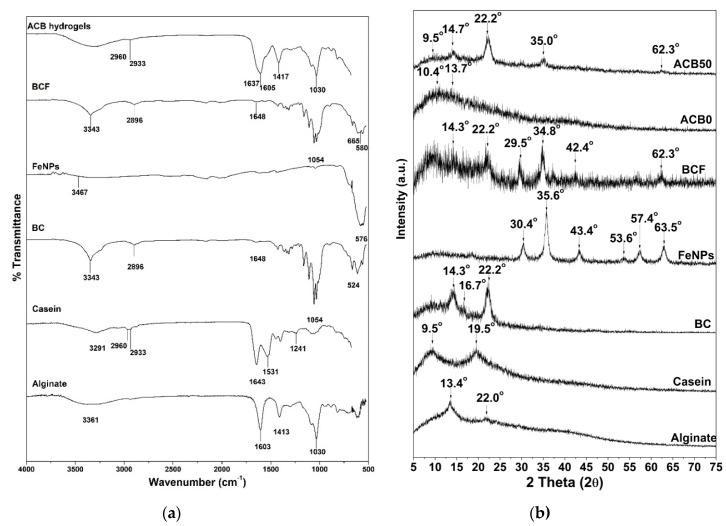
(**a**) Attenuated total reflectance (ATR) mode FTIR spectra and (**b**) X-ray diffractograms of synthesized FeNPs, BCF, free-dried ACB hydrogels, casein and alginate biopolymers.

FTIR of synthesized FeNPs, BCF, free-dried ACB hydrogels, casein and alginate biopolymers are presented in Figure 4a. IR studies were conducted to determine the formation of magnetite nanoparticles and casein proteins and to observe the changes in functional groups after the cross-linking reaction. The presence of the three specific amide bands, i.e., amide I, II and III at 1643, 1531 and 1241 cm^−1^, respectively, confirmed the formation of casein protein. Additionally, amide A and amide B bands at 3291 and 2960/2933 cm^−1^, respectively, were ascribed to –OH/N–H and –CH_3_ stretching vibrations, respectively [42]. Furthermore, FeNPs showed an absorption band at 576 cm^−1^ and a shoulder at 700 cm^−1^, attributed to the stretching and bending vibrations of the Fe−O, which ratified the formation of iron-oxide nanoparticles. The synthesized BCF showed the characteristic bands at 3343, 2896, 1648, 1054 and 665 cm^−1^, which were attributed to stretching vibration of the –CH group, bending of the –OH group and the –C–O–C– pyranose ring and –CH deformation of the cellulose I structure in BC, respectively. Notably, a peak at 576 and a shoulder at 700 cm^−1^ confirmed the presence of Fe_3_O_4_ NPs in BC. This confirmed the impregnation of FeNPs into the BC pellicle. Sharp and intense peaks at 3361, 1603, 1413 and 1030 cm^−1^ in the case of alginate were assigned to –OH, –COO (symmetric), –COO (asymmetric) and –C–O–C– stretching, respectively [43]. In ACB hydrogels, an amide B peak could be seen, similar to the casein sample. The peaks at 1633 and 1417 cm^−1^ could indicate carbonyl (–C=O) stretchings [43,44]. The disappearance of the NH_2_-related band at 1531 cm^−1^ and the appearance of a new band of amide at 1605 cm^−1^ confirmed the chemical cross-linking via amide linkages between the carboxylic groups of alginate and the amine groups of casein [43,45].

### 3.5. X-Ray Diffraction Analysis of Alginate–Casein-FeNP-Impregnated Bacterial Cellulose (ACB) Hydrogels

Figure 4b illustrates the X-ray diffractograms of synthesized FeNPs, BCF, free-dried ACB hydrogels, casein and alginate biopolymers. XRD diffractograms of both biopolymers, i.e., alginate and casein, revealed that they were semi-crystalline in nature. Alginate exhibited peaks at 2θ = 13.4° and 22.0°, while casein exhibited peaks at 2θ = 9.5° and 19.5°. Synthesized FeNPs showed characteristic peaks at 30.4° (220), 35.6° (311), 43.4° (400), 53.6° (422), 57.4° (511) and 63.5 (440), corresponding to Fe_3_O_4_ nanoparticles [1].

The synthesized BCF showed characteristic peaks for BC at 2θ = 14.3° (101), 16.7° (10$\bar{1}$) and 22.2° (002), respectively [34]. It is noteworthy to mention that all peaks specific to FeNPs could also be observed in BCF. However, there was a slight shift in these peaks, likely due to layering of BC nanofibers over the nanoparticles. The ACB0 gels showed a broad peak because of the combination of alginate and casein reflections at 2θ = 10.4° and 13.7°, respectively, which indicated proper mixing of both the biopolymers. For ACB50 hydrogels, the peaks became sharper with increased intensity, which indicated increases in crystallinity. Peaks were found at 2θ = 9.5°, 14.7°, 22.2°, 35° and 62.3°, which indicated the presence of casein, alginate, BC and FeNPs in the ACB hybrid hydrogels.

### 3.6. Compression Analysis of Prepared Alginate–Casein-FeNP-Impregnated Bacterial Cellulose (ACB) Hydrogels

**Figure 5:**
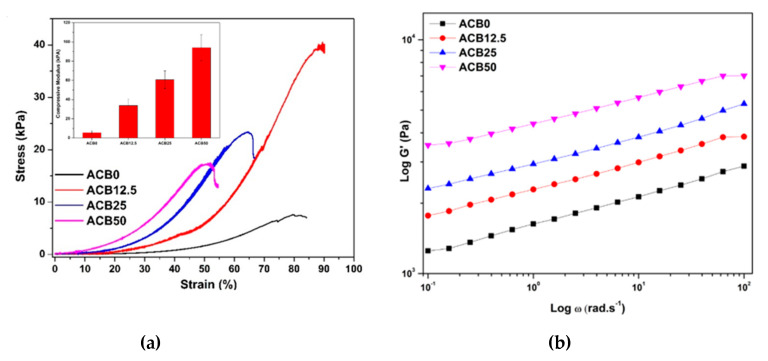

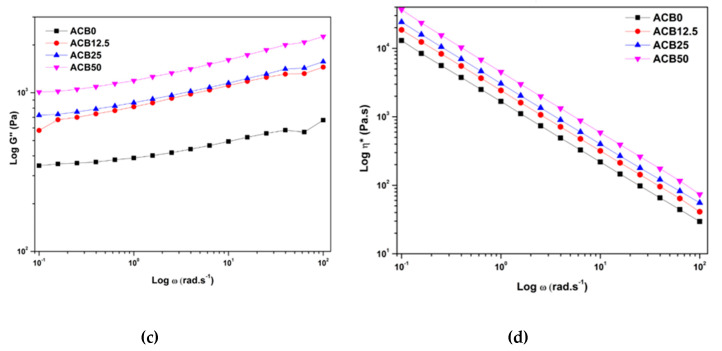
(**a**) Stress–strain profiles (inset: compressive modulus of elasticity), (**b**) storage modulus (G′), (**c**) loss modulus (G″) and (**d**) complex viscosity (η^*^) of swollen ACB hydrogels recorded at 25 °C.

**Figure 6:**
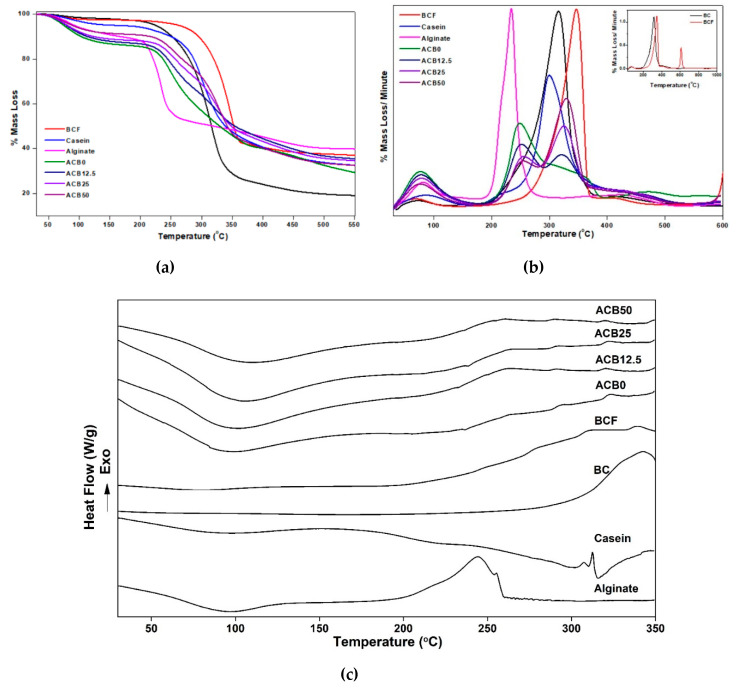
(**a**) Thermogravimetric (TG), (**b**) derivative TG and (**c**) DSC temperature profiles for alginate, casein, BC, BCF and ACB hydrogels.

The mechanical properties of cylindrical bulk ACB hydrogels were determined under uniaxial compression mode, and stress–deformation curves are presented as Figure 5a. The compression modulus of the ACB hydrogels are compared in Figure 6a (inset). From the stress–strain profiles as well as the compressive modulus, it is evident the incorporation of BCF into the cross-linked network greatly improved the compressive strength of the ACB hydrogels. The compression moduli were 5.6 ± 1.6, 33.8 ± 6.8, 60.7 ± 9.2 and 94.0 ± 3.6 kPa for ACB0, ACB12.5, ACB25 and ACB50, respectively. These results were in-line with the findings of Park et al., where oxidized BC (ca. 40 wt %) when incorporated into alginate hydrogels intended for cell encapsulation showed a compressive modulus of ~60 kPa [46]. Interestingly, in case of ACB50 and ACB25, BCF reinforced the cross-linked hydrogels up to about 55% strain. Beyond this strain limit, a series of fractures occurred that tended to weaken the gel structure. This could be corroborated with the SEM micrograph, where presence of agglomerates was observed. It can be concluded that above a 55% strain, the BCF network interacting with the hydrogel started to collapse. Similar finding were reported by Leon et al. [47].

### 3.7. Viscoelastic Behavior of Alginate–Casein-FeNP-Impregnated Bacterial Cellulose (ACB) Hydrogels

Oscillatory rheological studies were carried out on ACB hydrogels to understand the stability of three-dimensional cross-linked networks. As a typical hydrogel behavior, the storage modulus (G´) values of all ACB hydrogels was higher than loss modulus values (G´´), as shown in Figure 5b,c, over the entire range of angular frequency, i.e., from 0.1 to 100 rad·s^−1^ [14]. Due to reinforcement by the BCF network, the storage modulus of the hydrogels containing BCF was greater than alginate–casein hydrogels without BCF, which indicates improved cross-linking. At angular frequency (ω = 10 rad·s^−1^), storage modulus values were 2.1, 3.0, 3.8 and 5.7 kPa for 0, 12.5, 25 and 50 wt %, respectively, BCF-loaded alginate–casein hydrogels. Alginate-based hydrogels fabricated for tissue engineering applications can display improved storage modulus upon incorporation of fillers such as laponite clays, silver NPs, etc. [48,49]. The ACB50 had the highest values; the plausible reason for this could be formation of an intermolecular complex formation between chemically cross-linked hydrogels and the BCF network. Viscosity values (Figure 5d) were also increased upon incorporation of BCF, which indicates formation of a more rigid network inside the hydrogel systems.

### 3.8. Thermal Property Analysis of Prepared Alginate–Casein-FeNP-Impregnated Bacterial Cellulose (ACB) Hydrogels

Thermal stability is a key issue in choosing route for material processing. The thermal degradation profiles of alginate, casein, BCF and all ACB hydrogels were recorded from 25 to 600 °C under inert gas environment and are illustrated in Figure 6a. It can be seen that casein and BCF were thermally stable compared to alginate; therefore, the ACB hydrogels tended to degrade much later compared to alginate alone. In the derivative thermogravimetric profiles shown in Figure 6b, a broad peak from 50 to 150 °C was witnessed for all ACB hydrogels. This denotes the removal of bound water from the hydrogels. It is evident that incorporation of BCF improved the thermal stability of alginate–casein hydrogels. The ACB0 showed a degradation peak at ~247 °C, whereas ACB50 showed a degradation peak at ~255 °C. Shi et al. prepared BC/alginate hydrogels intended for drug delivery systems, and they found that BC has an important role in improving the thermal stability of BC/alginate hydrogels [50].

This degradation peak indicated the decomposition of polysaccharides chains [50]. The casein protein was found to degrade around 300 °C [51]. Upon mixing of alginate with casein, the resulting ACB0 hydrogels showed a delayed degradation over a longer range of temperature. Furthermore, incorporation of BCF resulted in single degradation peaks for both casein and BCF. However, they were shifted to higher temperatures. Similar results were obtained by subjecting the hydrogels and constituent biopolymers to DSC, as shown in Figure 6c. All hydrogels showed broad endothermic peaks in the range of 50 to 150 °C, which points towards the loss of moisture from the hydrogels. The degradation peaks for alginate, casein and BC could be seen as exothermic peaks at ~245, ~302 and ~342 °C, respectively. Interestingly, upon mixing the alginate with casein and BCF, the decomposition temperature shifted from ~245 to ~260 °C with one broad peak, suggesting compatibility between the constituents.

### 3.9. Magnetic Properties of Prepared Alginate–Casein-FeNP-Impregnated Bacterial Cellulose (ACB) Hydrogels

Magnetic response in hydrogels is much needed for directed drug delivery and cancer therapeutics.

**Figure 7:**
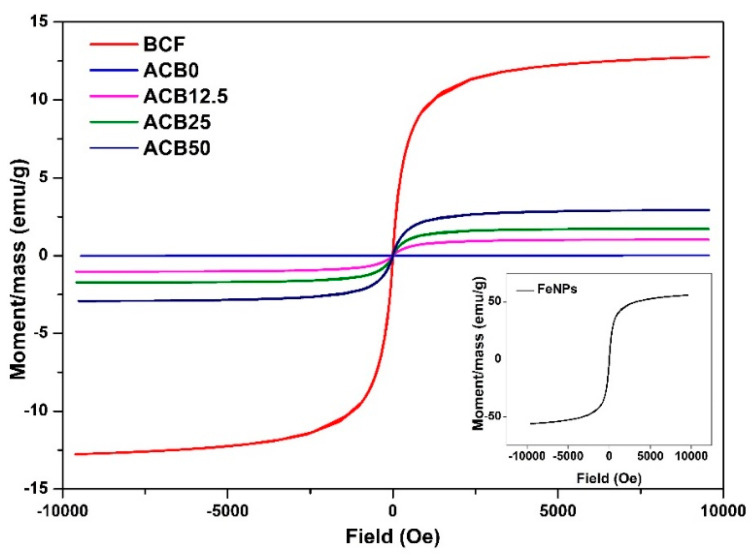
Magnetization curves for ACB hydrogels.

So as to investigate the magnetic behavior of the ACB hydrogels, hysteresis loops were acquired using a vibrating magnetometer and are presented in Figure 7. It can be seen that VSM curves for ACB hydrogels were identical in shape to those for BCF and FeNPs, which indicated that nanoparticles retained their intrinsic properties in the hybrid hydrogels [34]. It was observed that as the BCF content increased, the magnetic behavior was enhanced in the ACB hydrogels. The incorporation of BCF instead of the direct use of iron-oxide nanoparticles at such high concentrations made the hydrogels retain the magnetic character without compromising the dispersion and strength.

### 3.10. Drug Release Profiles of Prepared Alginate–Casein-FeNP-Impregnated Bacterial Cellulose (ACB) Hydrogels

**Figure 8:**
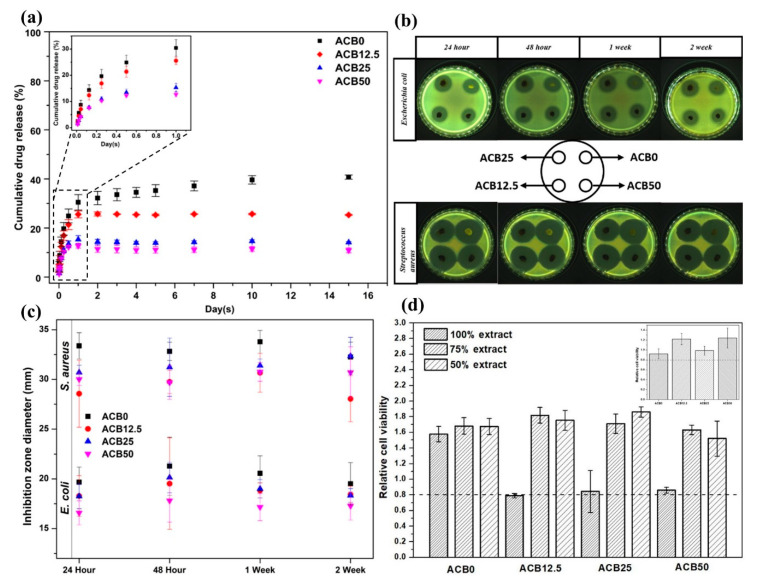
(**a**) Cumulative drug release, (**b**) antibacterial activity of ACB hydrogels, (**c**) inhibition zone diameters at 24 h, 48 h, 1 week and 2 weeks and (**d**) cytotoxicity of ACB hydrogels.

Ease of release of antibiotics is an essential requirement for treatment of burns or wounds [52]. The in vitro release of minocycline from ACB hybrid hydrogels was examined at human body temperature, i.e., 37 °C, for 15 days. Figure 8a presents the cumulative release of minocycline from ACB hydrogels. With the use of UV–vis spectroscopy, it was observed that all scaffolds had a loading efficiency of ~72%. It was observed that all ACB hydrogels demonstrated burst release for the initial 24 h and could reach 30.5, 25.8, 15.0 and 13.7% for ACB0, ACB12.5, ACB25 and ACB50, respectively. After initial burst release, not much release was observed for hydrogels incorporated with BCN. Sulaeva et al. reported similar findings, where alginate/BC wound dressings burst and released the entire antimicrobial agent within 48 hours of application [52].

This could be attributed to the presence of BCF, which promotes higher cross-linking, making the gel structure more compact, and therefore a longer diffusion path for the drug to diffuse out. Another possible reason could be binding of the drug with the hydrogel moieties. It can be concluded that ACB hydrogels display burst release for the initial 24 h, which is needed in cases of burn wounds, which are susceptible to infections and therefore require use of antibiotics. ACB hydrogels when injected into the dermis will serve as single application tissue templates, whereby they can supply not just drugs but also essential components such as growth factors, nutrients such as minerals and vitamins that would serve as treatments and growth signals, thus providing an environment favorable to cellular attachment and growth, and would degrade after a time period that is necessary for regeneration and re-regulation of the site of injury or burn. Furthermore, with the incorporation of BCF, the drug delivery dosage can be assisted and controlled by magnetic stimuli.

### 3.11. Antibacterial and Cytotoxicity Analysis of Prepared Alginate–Casein-FeNP-Impregnated Bacterial Cellulose (ACB) Hydrogels

The antibacterial efficacy of minocycline-loaded ACB hydrogels was evaluated. From Figure 8b, it can be seen that clear inhibition zones were achieved against the test strains, viz. *S. aureus* and *E. coli*, for all time points, i.e., 24 h, 48 h, 1 week and 2 weeks. This indicates that all ACB hydrogels were able to diffuse the antibiotics effectively in a sustained manner over long periods of time, which matches with the cumulative drug release profiles shown in Figure 8a. The ACB hydrogels were found to show higher inhibition against *S. aureus*. The inhibition zone diameters were found to be reduced upon incorporation of BCF, which increased cross-linking density in ACB hydrogels. The inhibition zone diameter for ACB0 and ACB50 for 2 weeks were 19.5 ± 2.1 and 17.2 ± 1.4, respectively, for *E. coli* and 32.2 ± 1.5 and 30.7 ± 2.6 for *S. aureus*, respectively, as shown in Figure 8c. Similarly, Sulaeva et al. used BC/alginate wound dressings, which lack innate antibacterial activity, and upon loading with poly(hexamethylene biguanide) hydrochloride was found to be effective against *E. coli* and *S. aureus* [52].

Considering the fact that the prepared injectable hydrogels are intended for biomedical applications, especially wound healing, the cytotoxicity of ACB hydrogels was evaluated. To investigate the cytotoxic effect of ACB hydrogels, murine embryonic fibroblasts were exposed to the scaffold extracts at different concentrations (100%, 75% and 50%).The MTT assay results shown in Figure 8d revealed that the cell viability for all ACB hydrogels with or without the presence of BCF were more than 80%, which indicated that ACB hydrogels did not show any cytotoxic effects. Moreover, the cell viability was higher compared to the reference. Furthermore, cell viability was tested in direct contact (Figure 8d, inset) to verify the results, and it was found that incorporation of magnetic BCF promoted cell-viability. Zhang et al. reported similar findings, where a BC/alginate/Zn^2+^ system was used for wound dressing applications. They found that zinc ions promoted cell viability [41]. Similarly, metal NPs were found to improve cell proliferation in silk-based biocomposites aimed for cancer therapy [1]. Thus, it can be concluded that the ACB hydrogels promote cell growth, which is desirable for wound healing applications. This indicates that the hydrogels have great potential for biomedical applications.

## 4. Conclusions

In summary, we developed hybrid alginate–casein hydrogels interpenetrated with bacterial cellulose impregnated with iron-oxide nanoparticles via EDC/NHS chemistry, ionic crosslinking of calcium ions and alginate, and supramolecular interaction with BCF. Casein provided the required biocompatibility, alginate allowed controlled gelation, and BCF reinforced the structure. ACB hydrogels were subjected to detailed and systemic characterization, viz. morphological, structural, thermal, mechanical, magnetic and rheological using SEM, FTIR, XRD, TGA, DSC, UTM, VSM and a rheometer to obtain material property information. The amide cross-linking between casein and alginate was proven using FTIR. The results showed that incorporation of BCF was significant in achieving tunable properties in hydrogels. Morphological characterization suggested that iron-oxide nanoparticles were impregnated within the bacterial cellulose network with ~20 wt % loading. The presence of BCF make pores rougher, which is essential for cell adhesion. Swelling studies showed that the hydrogels were fast swelling with high water uptake (~4000%), making them suitable wound dressing materials. Thermal analysis revealed that BCF makes hydrogels more thermally stable. Compressive tests revealed that ACB hydrogels had high compressive moduli and were mechanically more stable compared to gels without BC. The drug release profiles showed a sustained release rate over longer durations, which could be tuned with magnetic stimuli. In vitro cytotoxicity assays on the filled and un-filled ACB hydrogels showed significant cell viability during a culture time of 24 h. However, further studies on cytocompatibility using human cell lines will be carried out. Based on superior mechanical and magnetic properties, favorable cell viability and drug release, the said hybrid hydrogels could be applicable as injectable hydrogels and potential biomaterials for use as transdermal drug delivery system/wound healing and tissue regeneration, etc.

## Figures and Tables

**Table 1:** Nomenclature and composition of prepared Alginate–Casein–BCF (ACB) hydrogels.

Sample	Alginate (% *w/v*)	Casein (% *w/v*)	BCF (% *w/w*)
ACB0	2	2	-
ACB12.5	2	2	12.5
ACB25	2	2	25
ACB50	2	2	50
